# Carpal Tunnel Decompression Under Wide Awake Local Anaesthesia No Tourniquet Technique (WALANT): A Cost Effective and Outcome Analysis

**DOI:** 10.7759/cureus.42125

**Published:** 2023-07-19

**Authors:** Ahmad Faraz, Anisha Bahl, Shoaib Khan, Mahmood Ahmad, Mohammad N Khan, Syed Mannan, Jayachandaran Jayadeep, Krishna Kumar

**Affiliations:** 1 Trauma and Orthopaedics, North Cumbria Integrated Care, Carlisle, GBR; 2 Trauma and Orthopaedics, University of Central Lancashire, Carlisle, GBR; 3 Trauma and Orthopaedics, Whiston Hospital, Liverpool, GBR; 4 Trauma and Orthopaedics, Redcliffe Hospital, Oxford, GBR; 5 Trauma and Orthopaedics, Royal Victoria Hospital, Belfast, GBR; 6 Trauma and Orthopaedics, Cumberland Infirmary, Carlisle, GBR

**Keywords:** clinician-measured outcomes, cost effectiveness analysis, orthopaedic hand surgery, carpal tunnel decompression, walant

## Abstract

Introduction

Wide-awake local anaesthesia with no tourniquet (WALANT) technique is cost-effective, resource-friendly, and safe. This can be used as an alternative to hand surgery procedures in outpatient units. It can be performed in clinics or operating rooms.

Methods

We retrospectively evaluated the outcomes of WALANT for carpal tunnel decompression (CTD) over two years. Measured results include wound infections, relief of symptoms, paraesthesia, haematoma, Visual Analogue Scale (VAS), hospital anxiety and depression scale score (HADS) and cost-effectiveness.

Results

Eighteen patients underwent CTD under the WALANT technique over two years. VAS score was recorded at 3.1 ± 1.2 during the procedure and 1.67 ± 0.933 at two weeks follow-up. Persistent paraesthesia was found in only one patient at follow-up. Minimal bleeding was recorded during the procedure. No wound infections, revision surgery or post-operative haematoma formation were found. Hospital Anxiety and Depression Scale (HADS) was reported as 4.77 ± 2.1 after surgery. WALANT was also cost-effective, with an overall amount of £20.

Conclusion

Performing carpal tunnel decompression under WALANT in one stop upper limb clinic is a safe and cost-effective technique with no significant patient-related complications.

## Introduction

Wide-awake local anaesthesia no tourniquet (WALANT) is a surgical technique which utilises local anaesthetic and vasoconstricting agents to form a haemostatic effect, eliminating the need for a tourniquet and sedation [1]. Canadian plastic hand surgeon Dr Lalonde first implemented WALANT to decrease waiting time for surgery. Carpal tunnel syndrome (CTS) involves the compression of the median nerve within the carpal tunnel, causing pain, discomfort, numbness, tingling and weakness in the hand [2]. Conservative management includes the use of a splint, lifestyle changes, analgesics, and steroid injections to ease inflammation. Patients who fail conservative treatment undergo carpal tunnel decompression. Carpal tunnel decompression involves the relief of the median nerve by cutting through the transverse carpal ligament [3]. WALANT involves the use of epinephrine and lidocaine; epinephrine promotes hemostasis while lidocaine acts as an anaesthetic, eliminating the need for a tourniquet. WALANT offers diverse applications in hand surgery. It is described for use in tendon repair, grafting, grafting, tendon fusion, open reduction and internal fixation of hand fractures, arthrodesis, joint arthroplasty, Dupuytren's contracture and carpal tunnel release with satisfactory outcomes [4,5]. Gunasagaran et al. found that Visual Analogue Scale (VAS) scores of patients were reported higher in the tourniquet group [6]. 

Carpal tunnel decompression (CTD) under WALANT can be performed in upper limb clinics or office settings as a day-case procedure [7]. We aim to analyse the outcomes, cost-effectiveness and safety of performing WALANT in clinics.

## Materials and methods

Study design

This is a single-centred, single-surgeon, retrospective study conducted at a district general hospital involving 18 patients who underwent carpal tunnel decompression in an office setting utilising the WALANT technique from February 2021 and November 2022. Patients were categorised on the basis of age, gender, BMI and American Society of Anesthesiologists (ASA) grades. Exclusion criteria consisted of patients who previously had carpal tunnel decompression (revision surgery), bilateral carpal tunnel syndromes and a few other patients with contraindications to WALANT such as bleeding tendency, abnormal clotting profile, hypersensitivity to lidocaine, needle-phobia and anxious patients. Demographic data, including age, gender, BMI, and co-morbidities, were collected from electronic care records and a theatre database. Additionally, procedural information was obtained, including procedure site and length of operation, blood loss amount of local anaesthesia used, turnover time, and cost of instruments utilised. To further assess procedure outcomes such as analyse anxiety, patient convenience and pain, we used the Hospital Anxiety and Depression Scale (HADS) and the Visual Analogue Scale (VAS) scores as documented in clinic letters. VAS score is a spectrum of pain (from no pain to worst pain) to allow patients to classify the severity of the pain [8]. The HADS questionnaire evaluates anxiety levels, particularly regarding surgery in this context. There are 14 questions which can result in a score between 0 and 21 that allocates the patient's anxiety within four categories: normal (0-7), mild (8-10), moderate (11-15), and severe (16-21) [9]. Cost-effectiveness was calculated by the cost of resources used per patient when completing a CTD using WALANT compared to a traditional surgical theatre approach with an intraoperative tourniquet.

Clinic-based procedure and operative-setup

**Figure 1:**
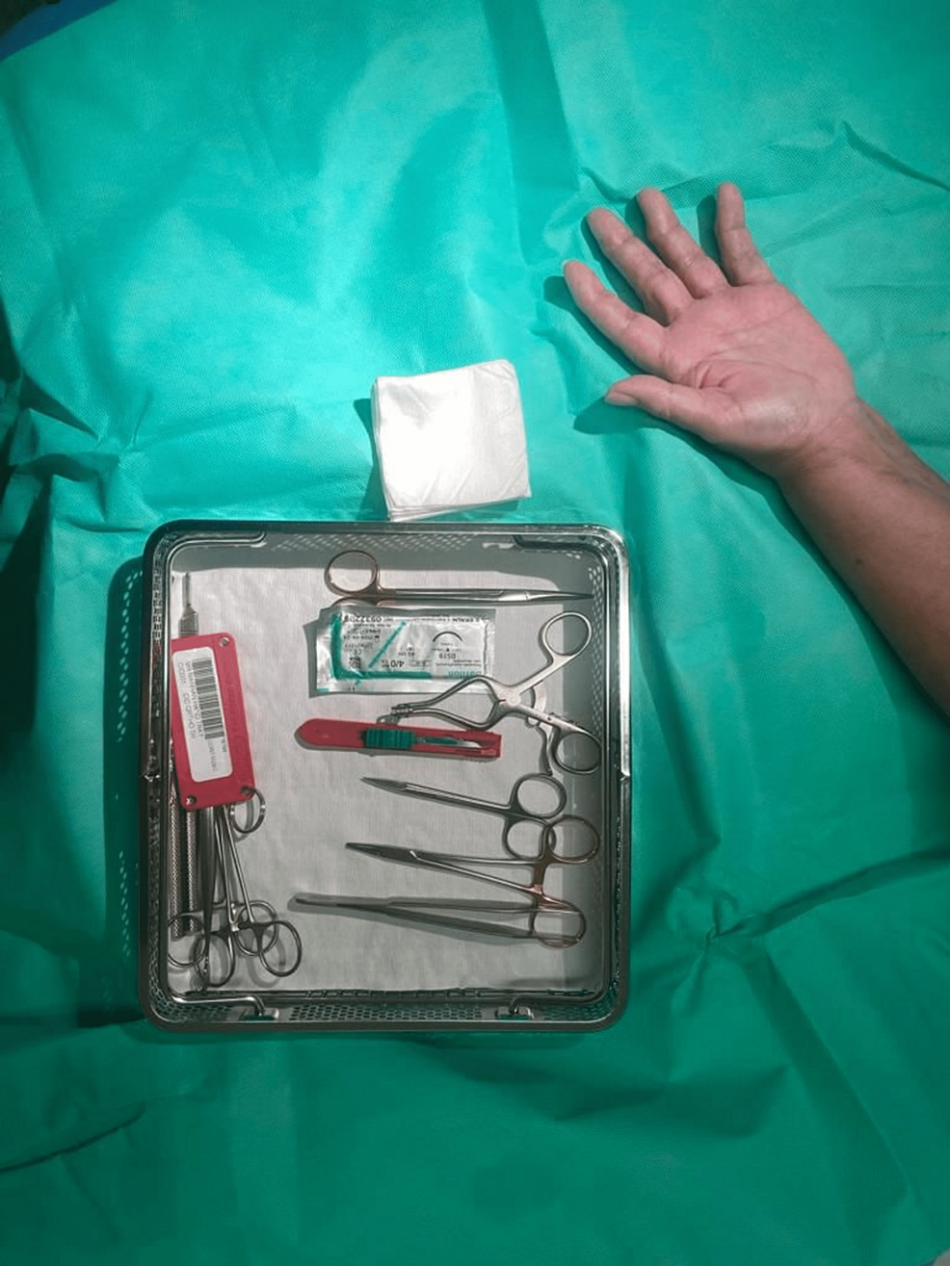
Carpal tunnel decompression kit

WALANT has been widely used in hand surgery procedures. This study aims to analyse the use of WALANT for CTD in upper limb clinics. We aim to highlight that CTR can be safely performed in office settings with the availability of a CTD kit, sterile gloves and drape. This technique proved to be cost-effective when compared with theatre session procedure which requires a sterile environment, multiple staffing, head covers, neck-to-knee sterile surgeon gowns, sterile patient drape shoe covers, and laminar airflow. The instruments required are shown in Figure 1. Our procedure room had the surgeon and patient sitting opposite to each other with the patient's hand lying on the table with a sterile drape. The patient was checked regarding comfort and pain during the procedure repeatedly.

Surgical WALANT technique

Patients undergoing CTR with WALANT were explained and consented about the procedure in detail. They were warned about multiple pricks for the infiltration of local anaesthesia. After obtaining formal consent, patients were prepped with a sterile drape for surgery. The patient underwent subcutaneous injection with 8-10ml of local anaesthetic (1% xylocaine and adrenaline), while the surgeon waited for haemostatic effect, and the procedure started within five minutes of administration of local anaesthetic. The procedure was carried out using the traditional CTR approach. An incision measuring 2 inches is made on the volar aspect of the hand, exposing the transverse carpal ligament, which is the roof of the carpal tunnel. The vacuum was further created under the tunnel, alleviating pressure on the median nerve. The incision was closed with non-absorbable sutures, and the hand was bandaged with Mepore (Mölnlycke Health Care, Gothenburg, Sweden), and woolen and crepe bandages.

Post-operative care

This procedure was performed as daycare; patients were assessed with vitals and observations for one hour post-operatively. They were discharged home without any antibiotics prescription; however, with appropriate analgesics such as paracetamol, ibuprofen and codeine phosphate. Patients were advised to observe for any wound discharge, digital ischemia, or complex regional pain syndrome. They were instructed to follow up in clinics after two weeks for wound review and removal of sutures; a thorough clinical assessment was performed and documented through digital clinic letters. VAS and HAD score was documented in the letter confirming the appropriateness of the procedure. Patients were discharged from care and were advised to undergo physiotherapy.

Cost comparison

Direct cost analysis was made with regard to instruments used in the procedure. The carpal tunnel decompression kit is shown in Figure 1.

## Results

Between February 2021 and November 2022, 18 patients underwent carpal tunnel release surgery in the one-stop upper limb clinic using WALANT. Demographic data noted from the patient group included age, gender, body mass index, smoking status, and the American Society of Anaesthesiologists (ASA) classification. There were 10 males and eight females involved in the study, whereas the mean age was 58 ± 6.2. Further data is shown in Table 1.

**Table 1:** Demographic data ASA - American Society of Anesthesiologists

Variables	Number of patients
Age	58 ± 6.2
Male	10
Female	8
BMI	28 ± 4.2
Smoke	4
ASA I	11
ASA II	7

Patients were given 8-10ml of 1% xylocaine and adrenaline prior to surgical commencement.VAS score and HADS were assessed after the procedure and at the two-week clinic visit. The results, alongside local anaesthetic dosage administered per patient, are outlined in Table 2. Mean VAS scores during the procedure and after two weeks were calculated as three and two, respectively. The VAS scores were rated between one and 10, where 10 was classed as the most severe pain experienced. The mean score confirmed minimal procedural pain despite the carpal tunnel release only being conducted with the addition of local anaesthesia for symptom relief, suggesting adequate patient comfort undergoing WALANT with the surgery in the office. The HADS can be scored between 0-21, where 21 is considered the most severe form of anxiety. HADS was 4.7 among the 18 patients assessed, advising that the majority of patients categorized their anxiety levels around the procedure as normal. 

**Table 2:** VAS score and HADS of 17 patients and their respective local anaesthetic dose VAS - Visual Analogue Scale, HADS - Hospital Anxiety and Depression Scale

	Local anaesthetic	Visual Analogue Scale (during procedure)	Visual Analogue Scale after two weeks	Hospital Anxiety Depression Scale
Patient 1	10mls (1% xylocaine and adrenaline)	2	1	5
Patient 2	6mls (1% xylocaine and adrenaline)	4	2	9
Patient 3	8mls (1% xylocaine and adrenaline)	3	1	3
Patient 4	5mls (1% xylocaine and adrenaline)	3	2	4
Patient 5	10mls (1% xylocaine and adrenaline)	3	1	8
Patient 6	6mls (1% xylocaine and adrenaline)	1	4	1
Patient 7	6.8mls (1% xylocaine and adrenaline)	8	2	5
Patient 8	9mls (1% xylocaine and adrenaline)	2	1	2
Patient 9	10mls (1% xylocaine and adrenaline)	1	3	3
Patient 10	8mls (1% xylocaine and adrenaline)	7	1	4
Patient 11	10mls (1% xylocaine and adrenaline)	1	2	2
Patient 12	10mls (1% xylocaine and adrenaline)	5	1	4
Patient 13	9mls (1% xylocaine and adrenaline)	6	2	1
Patient 14	10mls (1% xylocaine and adrenaline)	2	3	3
Patient 15	7mls (1% xylocaine and adrenaline)	3	1	6
Patient 16	6mls (1% xylocaine and adrenaline)	3	2	8
Patient 17	10mls (1% xylocaine and adrenaline)	1	1	8
Patient 18	7mls (1% xylocaine and adrenaline)	1	1	8

Common complications that can follow carpal tunnel decompression surgery include wound infections, haematomas, persistent paraesthesia, complex regional pain syndrome or the requirement for revision carpal tunnel decompression surgery. Given the 18 patients involved, none reported infection, and only one patient had persistent paraesthesia, which settled following the next clinic visit. 

In the assessment of the overall procedural cost associated with the equipment used when hosting the procedure in the clinic, it was noted that the collective cost was around £20, which provides a clear indicator of affordability and cost-effectiveness when compared to the costs corresponding to the traditional operating room (OR) approach. The tools and equipment prepared and used for each procedure are listed in Table 4, alongside their cost to obtain.

**Table 3:** Cost and equipment used per procedure

Equipment	Cost
Carpal tunnel decompression kit	15£
Local anaesthetic	0.10£
Syringes	1.10£
Ethilone	1.08 £
Mepore dressing	1.50 £
Wool and crepe	1.20 £

## Discussion

Performing theatre-based surgeries as an outpatient procedure in the clinic has been popular in health care, while many operations require sterility, access to crucial equipment, and a fully staffed theatre. However, certain specialities have found that field sterility in an ambulatory care setting is just as effective in maintaining good patient outcomes. The WALANT technique offers satisfactory outcomes and cost-efficiency in different hand and wrist conditions [10].

Previously, carpal tunnel release was conventionally thought to require a theatre to ensure success; having them done in the office has proven to be just as effective in providing optimal patient outcomes. Tulipan et al. found that patient satisfaction was 96% and 94% three months post-operative between the groups who underwent WALANT and conventional sedation, respectively. These percentiles deemed no significant statistical difference in outcomes between the two treatment groups [11]. Wellington et al. recorded favourable trends towards using WALANT versus monitored anaesthesia [12]. Post-operative care time was reduced when using WALANT compared to the other two methods. However, the complication rates did not vary between the techniques used, further supporting that WALANT can be used to perform the procedure safely [11]. Given the statistical support of satisfactory patient outcomes, WALANT's ability to safely and effectively decompress the carpal tunnel in an office setting can potentially reduce surgical caseload during times of high patient admittance. However, by expanding the environment by which elective surgeries like carpal tunnel syndrome treatment can be done, they do not have to be cancelled or delayed, especially in circumstances like COVID-19 [13].

VAS and HADS scores were obtained and analysed to assess the overall patient experience for those in the study who underwent carpal tunnel release surgery using WALANT in the upper limb clinic. This study recorded VAS scores during the procedure, and two weeks post-operatively, showing a low level of pain experienced by the 18 patients assessed, despite no sedation or operating room being used. Likewise, Tulipan et al. stated their average patient VAS scores at the two-week checkpoint were 2.32 for the WALANT group and 1.8 for the sedated group. Although pain after two weeks was slightly better for the patients who did not undergo WALANT, after three months, no significant difference was recorded among the two groups [11]. The mean HADS score for this study was 4.77, which displays minimal anxiety and a higher satisfaction rate towards the WALANT technique and its use in carpal tunnel decompression. Patients usually have higher preoperative anxiety and fear associated with the type of anaesthesia used for their procedure, with general anaesthesia generating anxiousness [14]. 

Cost-effectiveness is one of the crucial aspects that favour the WALANT technique in an office setting over the theatre. Specifically, it can eliminate the cost of a sterile operating room. Each patient cost £420 kit to perform the carpal tunnel release. When performed in an ambulatory setting, costs were estimated to be ¼ the amount needed for the same procedure to be done in a theatre. Kazmer et al. found that there was a drastic minimisation of the cost associated with performing WALANT in an ambulatory environment versus the operative room [15]. If this procedure is commonly implemented in upper limb clinics using WALANT, costs towards the National Health Services would drastically reduce. 

There are a few limitations to this study, which include being single-centred and having a smaller sample size, which may not be relative to the greater population in terms of outcomes. It is a retrospective study with a short follow-up time which can mask long-term outcomes and complications. There was no comparison group in our study. Future prospective studies and randomised control trials for various hand surgery procedures, along with a comparison of the groups, can be conducted to strengthen the evidence for the WALANT technique. However, despite this, we firmly believe that our study adds evidence to the literature published in support of WALANT for hand surgery procedures; it enlightens the cost-effectiveness and outcomes of performing hand surgery procedures in office settings.

## Conclusions

Wide-awake local anaesthesia no tourniquet (WALANT) is an alternative to general anaesthesia and tourniquet for undergoing surgery, requiring a minimal dose for procedures. This study concludes the fact that the WALANT technique for carpal tunnel release surgery has proven to be a safe and effective technique when performed in upper limb clinic procedure rooms, with reduced cost, limited delay, and satisfactory patient outcomes. Our study has shown no complications in short-term follow-up and has been found to be cost-effective.
